# The invasion process of the entomopathogenic fungus *Ophiocordyceps sinensis* into the larvae of ghost moths (*Thitarodes xiaojinensis*) using a GFP-labeled strain

**DOI:** 10.3389/fmicb.2022.974323

**Published:** 2022-09-02

**Authors:** Peipei Wu, Qilian Qin, Jihong Zhang, Huan Zhang, Xuan Li, Hongtuo Wang, Qian Meng

**Affiliations:** ^1^State Key Laboratory of Integrated Management of Pest Insects and Rodents, Institute of Zoology, Chinese Academy of Sciences, Beijing, China; ^2^College of Life Sciences, University of Chinese Academy of Sciences, Beijing, China

**Keywords:** Chinese cordyceps, entomopathogenic fungi, three-dimensional structure, infection, tegument invasion

## Abstract

Chinese cordyceps is a well-known and valuable traditional Chinese medicine that forms after *Ophiocordyceps sinensis* parasitizes ghost moth larvae. The low natural infection rate of *O. sinensis* limits large-scale artificial cultivation of Chinese cordyceps, and the invasion process is unclear. To investigate the temporal and spatial regulation when *O. sinensis* enters ghost moths, we constructed an *O. sinensis* transformant that stably expresses green fluorescent protein (GFP). Inoculating *Thitarodes xiaojinensis* larvae with a high concentration of GFP-labeled *O. sinensis*, we observed that *O. sinensis* conidia could adhere to the host cuticle within 2 days, germinate penetration pegs within 4 days, and produce blastospores in the host hemocoel within 6 days. The reconstructed three-dimensional (3D) structures of the invasion sites showed that penetration pegs germinated directly from *O. sinensis* conidia at the joining site with the larval cuticle. Differentiated appressoria or hyphae along the host epicuticle are not required for *O. sinensis* to invade ghost moths. Overall, the specific invasion process of *O. sinensis* into its host is clarified, and we provided a new perspective on the invasion process of entomopathogenic fungi.

## Introduction

Chinese cordyceps is a well-known traditional Chinese medicine with multiple pharmacological effects, including antitumor (Rao et al., 2007), immunomodulatory (Yang et al., 2011; Qian et al., 2012; He et al., 2013; Wu et al., 2014), and antioxidative activities (Dong and Yao, 2008; Wang et al., 2015). This natural medicine resource is a parasitic complex of the entomopathogenic fungus, *Ophiocordyceps sinensis* (Berk.), and its host, ghost moth (Lepidoptera: Hepialidae) larvae (Cheng et al., 2007; Sung et al., 2007; Li et al., 2019). *O. sinensis* can infect host larvae for 5–12 months, during which the larvae develop normally, and no noticeable symptoms appear on the larval surfaces (Li et al., 2020; Meng et al., 2021; Wu et al., 2022). A symbiotic relationship seems to exist between *O. sinensis* and its host (Holliday and Cleaver, 2008). The alpine meadows on the Tibetan Plateau at an altitude interval of 3,000–5,000 m are the main natural distribution areas of Chinese cordyceps, resulting in strict living conditions for this fungus and host insects (Zhang et al., 2012). Although artificial cultivation of Chinese cordyceps has been achieved, there are still many problems in the artificial cultivation process, such as a low natural infection rate and unclear invasion process. Injection inoculations not only consume vast amounts of labor and material resources but also increase the mortality of ghost moth larvae due to mechanical wounding. Therefore, exploring how *O. sinensis* invades its host, and improving the natural infection rate is crucial for large-scale artificial cultivation of Chinese cordyceps. Based on comparative genomics, previous studies have speculated that *O. sinensis* probably enters its host through its spiracles or orally (Wang et al., 2016). It has also been suggested that both cuticular and intestinal invasion are possible (Li et al., 2016). However, conclusive evidence regarding how the *O. sinensis* enters ghost moth larvae has not been reported.

The typical entry route of entomopathogenic fungi is that conidia, the initial invasion structures, germinate along insect epicuticles and produce narrow pegs to penetrate insect cuticles. The conidia of *Metarhizium* spp. and *Beauveria bassiana* could germinate and differentiate appressoria before breaching the insect tegument (Leger et al., 1987; St Leger et al., 1989; Wang and St Leger, 2005; Güerri-Agulló et al., 2010; Huang et al., 2015; de Sousa et al., 2021). Nevertheless, appressoria are unnecessary for entomopathogenic fungi to penetrate insect cuticles. For example, *Verticillium lecanii* hyphae, rather than appressoria, germinated from conidia can secrete mucilage and produce narrow pegs to penetrate the cuticles of adult and larval thrips, *Frankliniella occidentalis* (Schreiter et al., 1994). Overall, appressoria and hyphae developed from fungal conidia on insect epicuticles could produce penetration pegs and invade host insects.

Scanning electron microscopy (SEM) and transmission electron microscopy (TEM) are generally used to investigate the invasion process of entomopathogenic fungi (Schreiter et al., 1994; Asensio et al., 2005; Güerri-Agulló et al., 2010; Lei et al., 2021). However, based only on the shapes of fungal cells, it is difficult for us to identify target fungi on the cuticular surface of host insects. Although it has been reported that the internal transcribed spacer of the nuclear ribosomal RNA sequence can be used to identify the *O. sinensis* fungus, the PCR amplification with species-specific primers cannot visually present the spatial dynamics when *O. sinensis* enters its hosts (Liu et al., 2017; Zhang et al., 2018). Therefore, fluorescent labeling of *O. sinensis* is desirable to quickly and accurately distinguish *O. sinensis* cells from other structures.

Transformed organisms expressing fluorescent proteins are valuable tools for interaction studies among pathogens and hosts (Sesma and Osbourn, 2004; Amnuaykanjanasin et al., 2013; Sun et al., 2015; Steentjes et al., 2021). These fluorescent organisms can be easily observed using fluorescence microscopes. Green fluorescent protein (GFP) has been widely used as a marker. The promoters of *EF1*α, *gpdA*, and *trpC* were reported to drive GFP constitutively expressed in multiple filamentous fungi (Lorang et al., 2001), which allows the transformants to steadily and continuously express GFP at different developmental stages. The effectiveness of *Agrobacterium tumefaciens*-mediated transformation (ATMT) as a genetic analysis tool has been confirmed in various filamentous fungi (Rho et al., 2001; dos Reis et al., 2004; Ji et al., 2010; Hooykaas et al., 2018). It has been demonstrated that the ATMT of *O. sinensis* is successful (Liu et al., 2020). However, a GFP-labeled *O. sinensis* strain has not been constructed due to the difficult cultivation of *O. sinensis*. Slow growth of the fungus at low temperatures compared to *A. tumefaciens* also increases the failure rate of *O. sinensis* transformation.

In this study, an *O. sinensis* transformant expressing GFP was generated by the ATMT method. This transformant was used to inoculate *Thitarodes xiaojinensis* larvae, a major host of *O. sinensis*, and *O. sinensis* attached to the host epicuticle was observed with a confocal laser scanning microscope at different time points. The three-dimensional (3D) structures of invasion sites showed that *O. sinensis* conidia could directly germinate penetration pegs to enter the host hemocoel. Our results confirmed that appressoria or hyphae on the surface of the larval cuticle are not required for *O. sinensis* to breach the ghost moth tegument.

## Materials and methods

### Fungi, insects, and culture conditions

The *O. sinensis* wild-type strain was isolated from the fruiting bodies of *O. sinensis* according to a previously described method (Li et al., 2020; Meng et al., 2021). *O. sinensis* was cultured in peptone potato dextrose agar (PPDA) plates (10 g of peptone, 200 g of potato, 20 g of glucose, 1.5 g of KH_2_PO_4_, 0.5 g MgSO_4_, 20 mg of vitamin B1, and 15 g of agar per 1 L) at 18°C for conidium formation and cultured in the medium (PPDA without agar) at 18°C for blastospore formation (Liu et al., 2018, 2020). The resulting fungal cultures were filtered with Miracloth (Millipore Sigma, USA) to harvest blastospores or conidia.

*T. xiaojinensis* was collected from Xiaojin County, Sichuan Province, and had been reared in our laboratory for several generations. Larvae were reared on carrots at 16 ± 1°C and 70% relative humidity.

### Inoculation

*O. sinensis* conidia were washed three times with 0.05% Tween-80 and were eventually suspended in 0.05% Tween-80 to a 4.0 × 10^7^ cells/ml concentration. Second-instar *T. xiaojinensis* larvae were immersed in 1 ml of conidium suspension for 10 s, and all treated larvae were then reared under normal conditions.

### Sample preparation of SEM

*T. xiaojinensis* larvae inoculated with *O. sinensis* conidia were fixed with 2.5% glutaraldehyde overnight at 4°C. The samples were then washed with 0.2 M phosphate buffer (PB, pH 7.4) and dehydrated through an ethanol gradient (e.g., 30, 50, 70, 80, 90, and 100%) for 45 min each. The final dehydration of 100% ethanol was divided into three rounds of 15 min each. After ethanol was displaced by liquid carbon dioxide, the samples were dried using a CPD030 critical point drying apparatus. After being sputtered with gold, the samples were observed using a Hitachi SU8000 series SEM (Hitachi, Japan).

### Incubation on hydrophobic interface

Incubation of *O. sinensis* conidia on the hydrophobic interface was performed as previously described (Wang and St Leger, 2007). Briefly, membranous locust wings were sterilized in 37% H_2_O_2_ for 5 min and washed two times in sterile water. Sterile wings were immersed in conidium suspensions (4.0 × 10^7^ cells/ml in 0.05% Tween-80) for 20 s and placed on 2% water agar plates for incubation at 18°C. Germination of *O. sinensis* conidia was consecutively observed for 16 days.

### Plasmid construction

**Figure 1:**
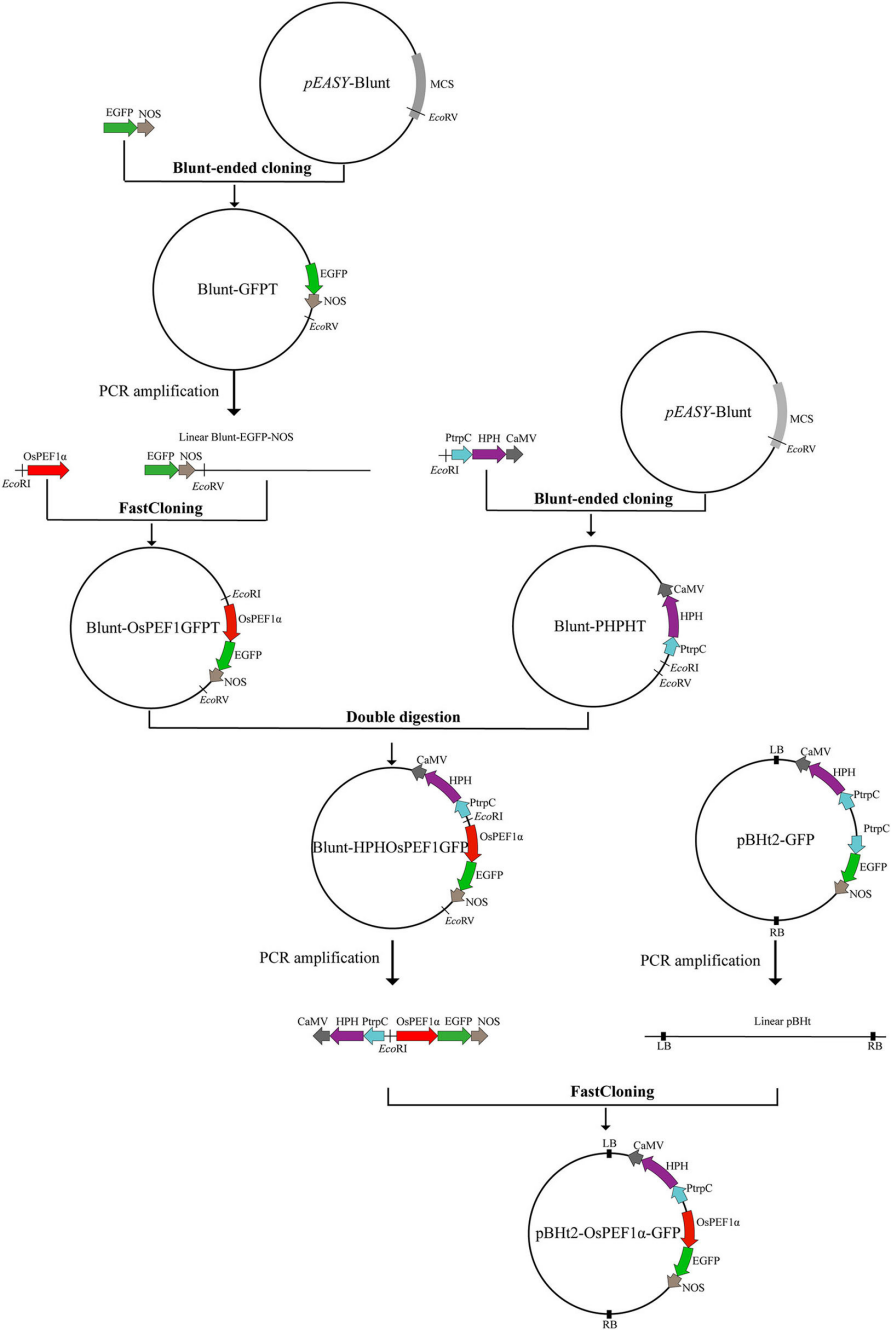
Flowchart for constructing plasmid pBHt2-OsPEF1α-GFP. Blunt-ended cloning, FastCloning, and restriction enzyme double digestion methods were used to construct the plasmid pBHt2-OsPEF1α-GFP. The *pEASY*-Blunt cloning vector is an intermediate vector. MCS, multiple cloning site. The plasmid pBHt2-OsPEF1α-GFP contains two gene expression cassettes: the *hygromycin B phosphotransferase enzyme* (*HPH*) gene cassette with the *Aspergillus nidulans trpC* promoter and *CaMV 35S* terminator and the *EGFP* gene cassette with the *Ophiocordyceps sinensis EF1*α promoter and *NOS* terminator.

The binary vector, pBHt2-GFP (purchased from BioVector, China), was confirmed by sequencing, which contained two gene expression cassettes: the *hygromycin B phosphotransferase enzyme* (*HPH*) gene cassette with the *Aspergillus nidulans trpC* promoter and *CaMV 35S* terminator and the *EGFP* gene cassette with *A. nidulans trpC* promoter and *NOS* terminator (Figure 1). *A. nidulans trpC* promoter ahead of *EGFP* was replaced by *O. sinensis EF1*α promoter (GenBank accession number: ON651447) to improve the expression efficiency of GFP, resulting in a new binary vector, pBHt2-OsPEF1α-GFP. Due to the lack of an available restriction enzyme site at opposite ends of the *A. nidulans trpC* promoter fragment in the pBHt2-GFP vector, the *pEASY*-Blunt cloning vector (TransGen Biotech, China) was used as an intermediate vector to construct a new vector, pBHt2-OsPEF1α-GFP, through blunt-ended cloning, FastCloning (Li et al., 2011), and restriction enzyme double digestion methods (Figure 1). The primers used for plasmid construction are listed in Supplementary Table 1.

### Fungal transformation

ATMT of *O. sinensis* was performed as previously described (Liu et al., 2020) with a slight modification. Briefly, the binary vector, pBHt2-OsPEF1α-GFP, was introduced into the *A. tumefaciens* AGL-1 strain (Biomed, China) by a heat-shock method according to the manufacturer's instructions. *A. tumefaciens* AGL-1 cells carrying pBHt2-OsPEF1α-GFP were cultured in the induction medium (dos Reis et al., 2004) containing 200 μM acetosyringone at 28°C, and the OD_660_ values ranged from 0.15 to 0.6. A 100-μl aliquot of preinduced AGL-1 cells was mixed with 100 μl of *O. sinensis* blastospores (1.0 × 10^7^ cells/ml), and the mixture (200 μl) was spread onto a cocultivation plate (dos Reis et al., 2004) and cultured for 72 h at 20°C. The cocultures were selected by PPDA plates containing 300 μg/ml hygromycin B and 300 μg/ml cefotaxime at 18°C until fungal colonies formed.

### Evaluation of transformant stability

The putative *O. sinensis* transformants were examined by fluorescence microscopy. Strong-green-fluorescence strains were transferred to PPDA plates and PPDA medium to generate conidia, blastospores, and hyphae. To evaluate the stability of the transformants, different formulations of GFP-labeled *O. sinensis* were observed with fluorescence microscopy. Furthermore, Chinese cordyceps that formed after the GFP-labeled *O. sinensis* strain infected *T. xiaojinensis* were checked with the IVIS Spectrum Imaging System (PerkinElmer, USA).

### Microscopic observation of the infection process

After 2, 4, and 6 days of inoculation, inoculated larvae were observed with a confocal laser scanning microscope (LSM710, ZEISS, Oberkochen, Germany) at excitation/emission wavelengths of 488/521 nm. At least 3 larvae were randomly selected for observation at each time point. To observe the complete fungal invasion structures, *z*-stack scanning was used to collect images at different specimen depths. Due to the autofluorescence of the *T. xiaojinensis* larval cuticle, excitation/emission wavelengths of 561/640 nm were used to exclude nonspecific fluorescent signals.

### Image processing

All images taken by the confocal laser scanning microscope were processed with Fiji software. Two-dimensional (2D) maximum intensity projections of *z*-stack fluorescent images were created to show the complete fungal structure from outside to inside the larvae. To enable more intuitive observations, 3D images of the invasion sites were reconstructed.

### Analysis of selected genes in different entomopathogenic fungal genomes

Genes encoding adhesins, hydrophobins, and key melanin-synthesis proteins were derived from published genomic data, including *O. sinensis* (Shu et al., 2020), *Cordyceps militaris* (Zheng et al., 2011), *B. bassiana* (Xiao et al., 2012), and *Metarhizium robertsii* (Hu et al., 2014), and then their numbers were counted.

## Results

### Appressoria were not observed from *Ophiocordyceps sinensis* conidia

After inoculation with *O. sinensis* conidia, we initially used SEM to observe the epicuticles of *T. xiaojinensis* larvae. We found *O. sinensis* conidia only on the epicuticles of ghost moths, without germinated structures such as appressoria and hyphae (Supplementary Figure 1). Diverse microorganisms and debris were present on the cuticular surfaces of ghost moths due to the complex living conditions of the larvae in soil. The incubation assay on the hydrophobic interface was also conducted. We found that *O. sinensis* conidia could germinate on the membranous wings of locust, but appressoria were not produced (Supplementary Figure 2). These results prompted us to speculate that appressoria were probably unnecessary when *O. sinensis* invaded its hosts.

### Stable green fluorescence was observed in *Ophiocordyceps sinensis* transformants

**Figure 2:**
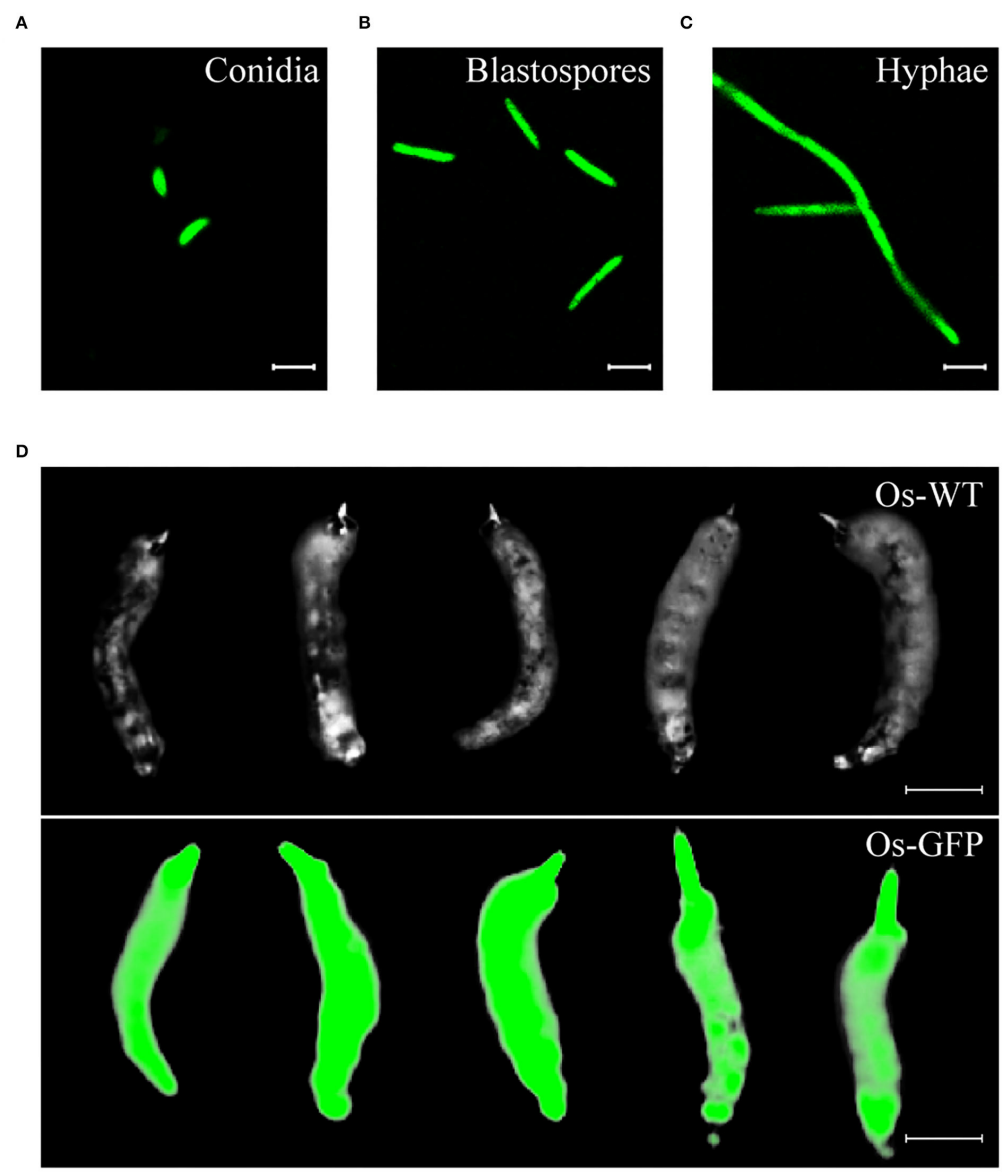
The *Ophiocordyceps sinensis* transformant generated with the pBHt2-OsPEF1α vector constitutively expressed green fluorescent protein. Conidia **(A)**, blastospores **(B)**, and hyphae **(C)** of the GFP-labeled *O. sinensis* strain were visualized at a 488-nm excitation wavelength. **(D)** Chinese cordyceps derived from the GFP-labeled *O. sinensis* strain still had a green fluorescent signal. Scale bars in **(A–C)**, 10 μm. Scale bar in **(D)**, 1 cm.

To investigate the invasion process of *O. sinensis* to its host insect, we constructed a GFP-labeled *O. sinensis* transformant. The binary vector pBHt2-OsPEF1α-GFP was used for ATMT of *O. sinensis*, and GFP was constitutively expressed in different formations of *O. sinensis* transformants, including conidia, blastospores, and hyphae (Figures 2A–C). GFP-labeled *O. sinensis* infected *T. xiaojinensis* larvae and eventually formed green-fluorescence Chinese cordyceps (Figure 2D). These results suggested that GFP-labeled *O. sinensis* could be used for researching the invasion process of *O. sinensis* into *T. xiaojinensis* larvae.

### *Ophiocordyceps sinensis* conidia longitudinally germinated and directly invaded *Thitarodes xiaojinensis* larvae through tegument

**Figure 3:**
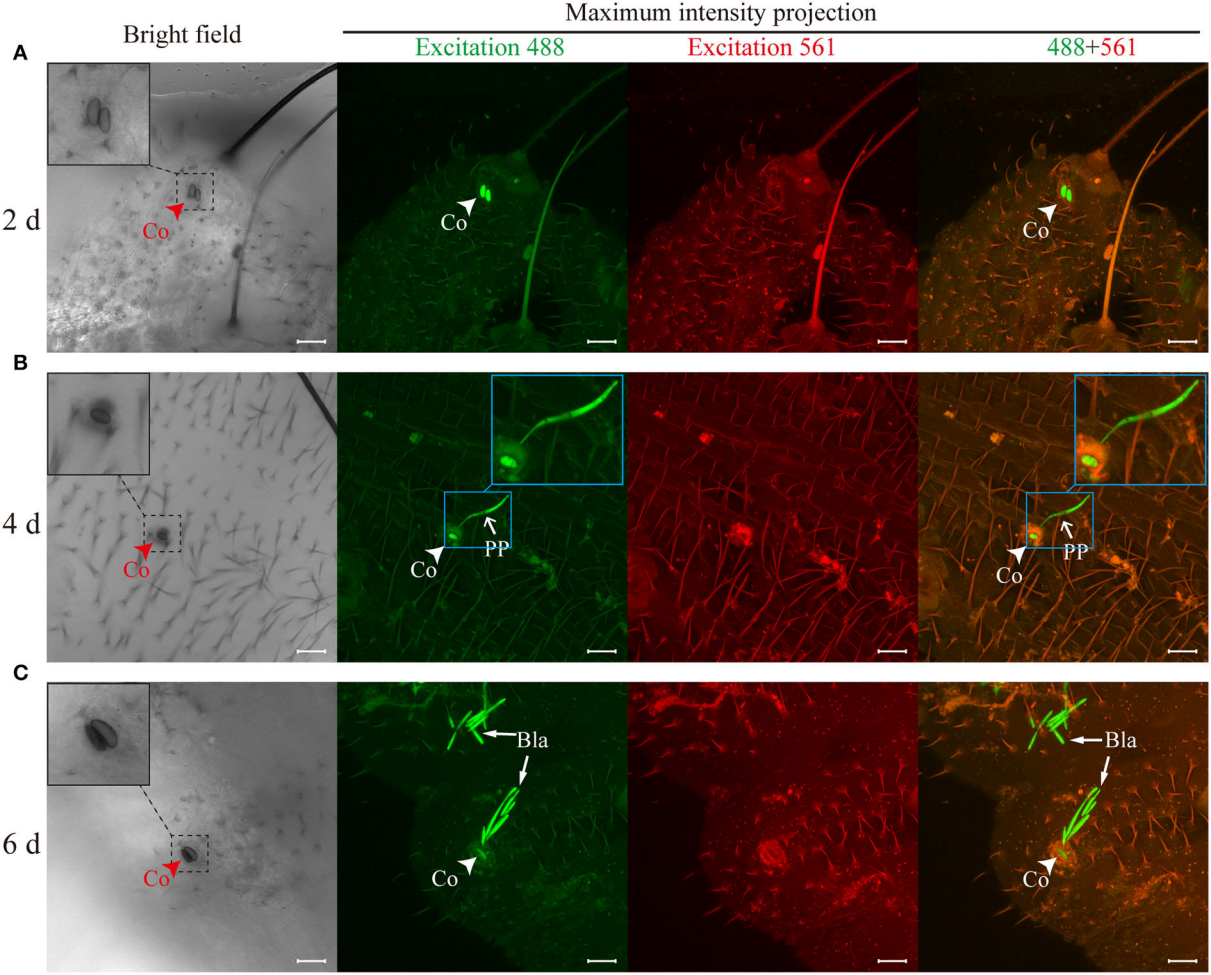
*Ophiocordyceps sinensis* invaded *Thitarodes xiaojinensis* larvae through teguments within 6 days. **(A)** Conidia attachment to the cuticles was observed after 2 days of inoculation. **(B)** The germ tubes penetrated the teguments and entered the larval body cavities after 4 days of inoculation. **(C)** Blastospores were observed in the body cavities of *T. xiaojinensis* larvae after 6 days of inoculation. The bright-field images were taken on the focal plane of the cuticle attached by conidia. Fluorescent images were maximum intensity projections of *z*-axial stacks. Green fluorescent images were taken at a 488-nm excitation wavelength. Red fluorescent images were taken at an excitation wavelength of 561 nm. In the merged image, the ghost moth cuticle is shown in orange due to the autofluorescence of the cuticle, and *O. sinensis* is specifically shown in green. Co, conidium; PP, penetration peg; Bla, blastospore. Scale bar, 20 μm. All photographs were taken by a confocal laser scanning microscope.

At different time points, *T. xiaojinensis* larvae inoculated with GFP-labeled *O. sinensis* were observed. At 2 days after inoculation, conidia attached to the epicuticle of *T. xiaojinensis* larvae (Figure 3A). The conidia could germinate tubes to penetrate the teguments and reach the larvae hemocoels after 4 days of inoculation (Figure 3B). Blastospores could be observed in the hemocoels of *T. xiaojinensis* larvae within 6 days postinoculation (Figure 3C). Regardless of which invasion period mentioned above was examined, conidial germination structures could not be observed on the focal plane of the epicuticle attached by the conidia (bright field in Figure 3). Penetration pegs and blastospores within the larvae hemocoels could be observed in fluorescent images that were composited from different tissue depths (maximum intensity projections in Figures 3B,C). Additionally, we observed melanized *O. sinensis* conidia when invading (bright field in Figure 3). Based on the annotation of published genomic data, we found that the number of genes involved in melanin synthesis in the *O. sinensis* genome was greater than those in *C. militaris, B. bassiana*, and *M. robertsii* (Supplementary Table 2). Taken together, *O. sinensis* could invade *T. xiaojinensis* larvae within 6 days.

**Figure 4:**
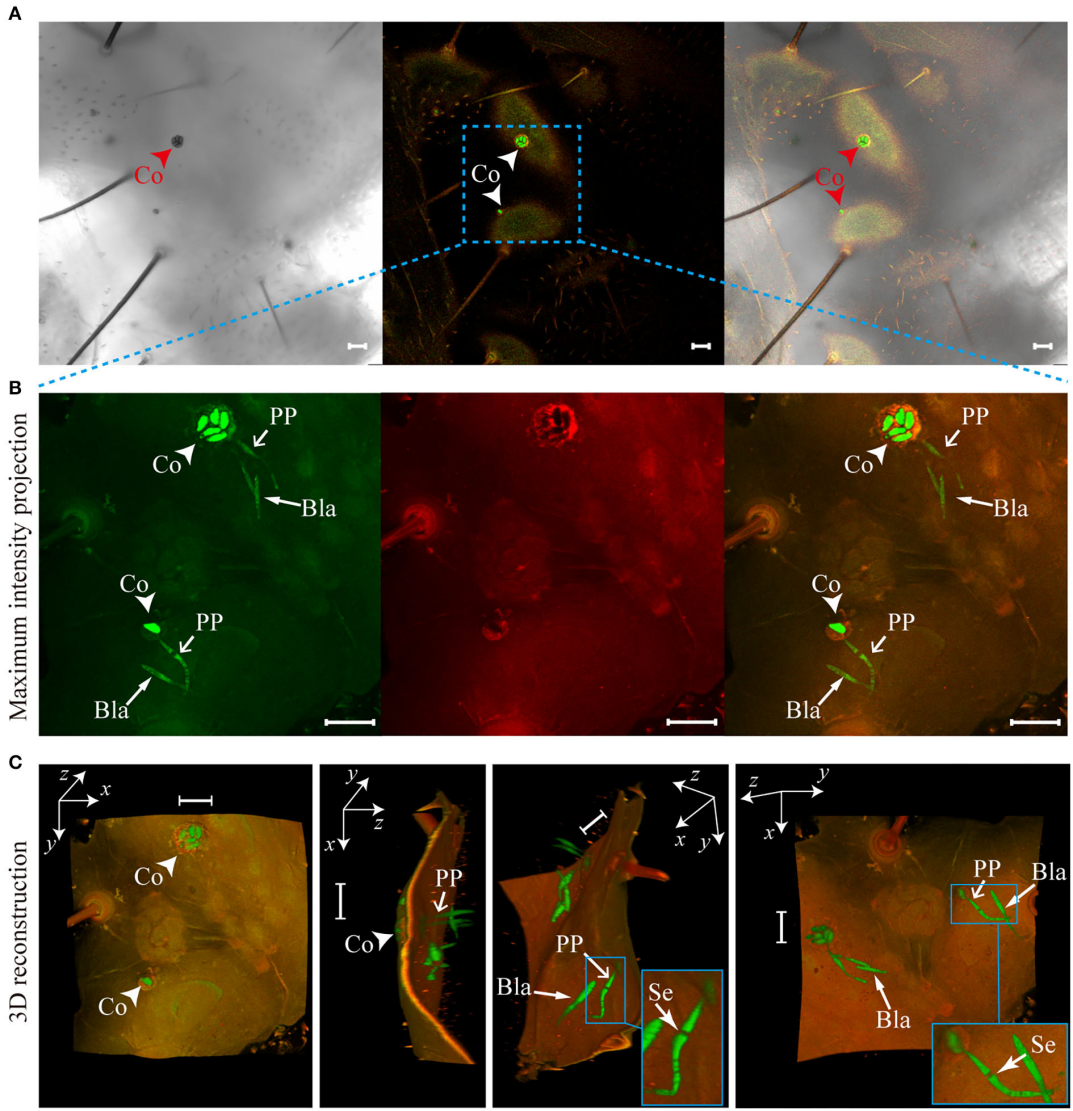
Penetration pegs directly derived from *Ophiocordyceps sinensis* conidia punctured the teguments of *Thitarodes xiaojinensis* larvae. **(A)** *O. sinensis* conidia on the epicuticles of *T. xiaojinensis* larvae. **(B)** Maximum intensity projections of the *z*-axial stacks [noted region in **(A)**]. **(C)** Three-dimensional (3D) reconstruction of the noted region in **(A)** using Fiji software. Co, conidium; PP, penetration peg; Se, septum; Bla, blastospore. Scale bar, 20 μm.

*O. sinensis* conidia directly digested the teguments of *T. xiaojinensis* larvae, and penetration pegs germinated from conidia could be clearly observed from the 3D reconstruction of invasion sites (Figure 4C, Supplementary Videos 1, 2). Additionally, specific sites, such as spiracles and mouthparts, were not required to invade host insects (Figures 3, 4). After reaching *T. xiaojinensis* larvae hemocoels, the invasion structures produced blastospores in the hemolymph, and septa were observed in the penetration pegs (Figure 4C, Supplementary Videos 1, 2). These results demonstrated that *O. sinensis* conidia, rather than appressoria or hyphae, directly germinated penetration pegs to invade larvae through the teguments.

## Discussion

This study found that *O. sinensis* conidia could invade *T. xiaojinensis* larvae through the host teguments, which was not previously reported for this fungus. Effective experimental materials and methods have contributed to revealing the invasion process of *O. sinensis* into its host. The GFP-labeled *O. sinensis* strain facilitated us in quickly finding target fungi on the surface of the *T. xiaojinensis* larval cuticle. The SEM observations in previous studies showed only the cuticular surfaces of host insects (Güerri-Agulló et al., 2010). Although TEM could show the invading structure of pathogenic fungi from outside to inside the host, it is difficult for us to identify which structures (conidia, appressoria, or hyphae) on the host epicuticle produce penetration pegs based on 2D section diagrams (Schreiter et al., 1994). In the current study, scanning certain thicknesses of larval tissue and reconstructing the 3D structure of the invasion sites clearly and altogether showed *O. sinensis* conidia on the host epicuticle and penetration pegs within the body cavity of *T. xiaojinensis* larvae. Therefore, labels with fluorescence and reconstructions of 3D structures are practical approaches to research the invasion process and even other interactions of pathogens with hosts.

A previous study speculated that *O. sinensis* was probably incapable of breaching host cuticles because of the absence or reduction of protein families involved in adhesion cuticles, appressorium formation, and cuticle degradation based on comparative genomic analysis (Hu et al., 2013). Comfortingly, our study is the first to unravel this mystery and provides solid evidence for tegument invasion by *O. sinensis*. Although the specific adhesion genes, *Mads*, were not found in the *O. sinensis* genome, four genes encoding hydrophobins in the *O. sinensis* genome may facilitate the attachment of *O. sinensis* conidia to insect cuticles (Zhang et al., 2011; Shu et al., 2020). According to our observations, appressoria were not produced from *O. sinensis* conidia on the surfaces of ghost moth cuticles and locust wings. The absence of appressoria on the host cuticle and hydrophobic interface was consistent with that of the 3D structures of invasion sites. An earlier study demonstrated that the *O. sinensis* serine proteases, Csp1 and Csp2, could degrade the cuticle proteins of *Hepialus* sp. larvae *in vitro* (Zhang et al., 2008). Based on the genomic analysis of *Metarhizium* spp. including wide-host-range species and narrow-host-range species, a study demonstrated that the gene families involved in fungal virulence in generalists are larger than those in specialists (Hu et al., 2014). Enriched fungal virulence genes facilitate generalist pathogenic fungi adaptation to different insect hosts. Nevertheless, *O. sinensis* exclusively infects ghost moth larvae (Shrestha et al., 2010), so redundant gene families are probably not required for invading a specific host. Overall, the reduced number of genes involved in adhesion cuticles, appressorium formation, and cuticle degradation is sufficient for *O. sinensis* to breach the cuticle of ghost moths.

Although the gene families related to adhesion cuticles, appressorium formation, and cuticle degradation are relatively reduced in *O. sinensis*, the invasion of *O. sinensis* into host larvae is indeed efficient. When *B. bassiana* or *Cordyceps fumosorosea* infects host insects, the less sclerotic regions of the host tegument, such as intersegmental folds or spiracles, are the preferred penetrating sites (Güerri-Agulló et al., 2010; Lei et al., 2021; Nithya et al., 2021). According to our observations, these particular invasion sites were not necessary for *O. sinensis* to enter ghost moth larvae. Based on SEM or TEM observations, a variety of entomopathogenic fungi first germinate appressoria or hyphae along the host epicuticle, and then appressoria or hyphae produce penetration pegs. For example, *B. bassiana* conidia germinate and differentiate appressoria or hyphae along the cuticle of red palm weevils with mucilage, which showed signs of penetration (Güerri-Agulló et al., 2010). Most *Metarhizium* were reported to form clearly defined appressoria, which produce hyphae that achieve penetration *via* a combination of mechanical pressure and hydrolytic enzymes (Ortiz-Urquiza and Keyhani, 2013, 2016). For instance, *Metarhizium anisopliae* and *Metarhizium humberi* conidia form short germ tubes, which swell to grow appressoria at the tips (St Leger et al., 1989; de Sousa et al., 2021). It has been reported that appressoria are essential for the invasion of most entomopathogenic fungi (Lin et al., 2011; Butt et al., 2016). However, the presence of appressoria and hyphae along the *T. xiaojinensis* larval cuticle surface was unnecessary for *O. sinensis* to invade hosts. We found that *O. sinensis* conidia could directly produce penetration pegs from the joining site of conidia with the host cuticle. Therefore, *O. sinensis* conidia as inoculation materials are feasible during the artificial cultivation of Chinese cordyceps, and increasing the amounts of conidia will likely promote the infection of *O. sinensis* to its hosts.

In addition to conidium adhesion and cuticle degradation, cell wall rigidity may influence the infection rate. It has been reported that melanin enhances the cell-wall rigidity of appressoria in the critical penetration phase of the plant pathogens, *Colletotrichum graminicola* and *Magnaporthe oryzae* (Horbach et al., 2009; Ludwig et al., 2014). In our study, melanized conidia were observed, and pigment deposition was notable as the invasion progressed. Moreover, gene families related to melanin synthesis in *O. sinensis* were larger than those in *C. militaris, B. bassiana*, and *M. robertsii*. Nevertheless, further research is required to determine whether melanin plays an essential role in tegument invasion.

## Conclusion

**Figure 5:**
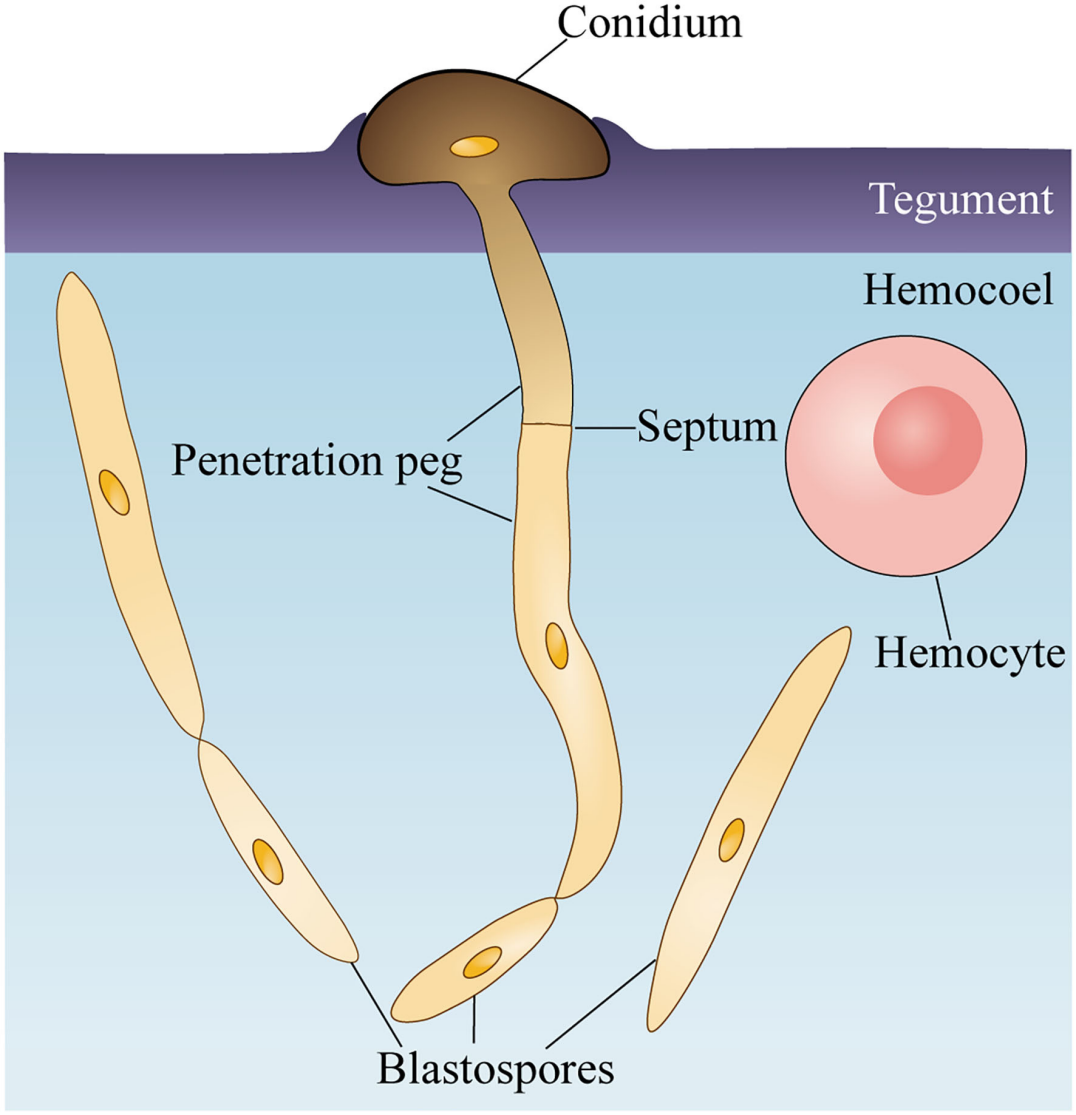
Schematic of *Ophiocordyceps sinensis* conidium invading *Thitarodes xiaojinensis* larvae. *O. sinensis* conidium attaches and degrades the teguments of *T. xiaojinensis* larvae. Germinated conidium directly produces a penetration peg to enter the hemocoel. After reaching *T. xiaojinensis* larvae hemocoels, the penetration peg generates a blastospore and proliferates in a yeast-type propagation strategy.

Taken together, *O. sinensis* could accomplish the invasion stage within 6 days and colonize *via* blastospores in the host hemocoel if a high concentration of conidium suspension was applied for inoculation. During the invasion stage, *O. sinensis* conidia attaching to the larval epicuticle germinated and directly produced penetration pegs from the joining site with the host cuticle surface (Figure 5). The discovery of the tegument invasion route provides a theoretical basis for large-scale artificial cultivation of Chinese cordyceps and new ideas for improving the infection rate of *O. sinensis*.

## Data availability statement

The data presented in the study are deposited in the GenBank repository, accession number ON651447. The data has been released.

## Author contributions

QM, QQ, and PW designed the research. PW and QM constructed the GFP strain. PW and QQ performed the inoculation and observation. PW processed the images and wrote the manuscript. JZ and HZ contributed to the study conception and the paper revision. XL and HW prepared the experimental insects and fungi. All authors contributed to the article and approved the submitted version.

## Funding

This work was supported by the National Natural Science Foundation of China (32000345, 31872297, and 31772525).

## Conflict of interest

The authors declare that the research was conducted in the absence of any commercial or financial relationships that could be construed as a potential conflict of interest.

## Publisher's note

All claims expressed in this article are solely those of the authors and do not necessarily represent those of their affiliated organizations, or those of the publisher, the editors and the reviewers. Any product that may be evaluated in this article, or claim that may be made by its manufacturer, is not guaranteed or endorsed by the publisher.
