# Supplementary material for: The invasion process of the entomopathogenic fungus Ophiocordyceps sinensis into the larvae of ghost moths (Thitarodes xiaojinensis) using a GFP-labeled strain
Source: Front Microbiol. 2022 Sep 2;13:974323. doi: 10.3389/fmicb.2022.974323 (PMC9479185; doi:10.3389/fmicb.2022.974323)
Supplement: Supplementary file 3 [file Data_Sheet_1.docx]

Supplementary Figures and Tables

# Supplementary Figures

**
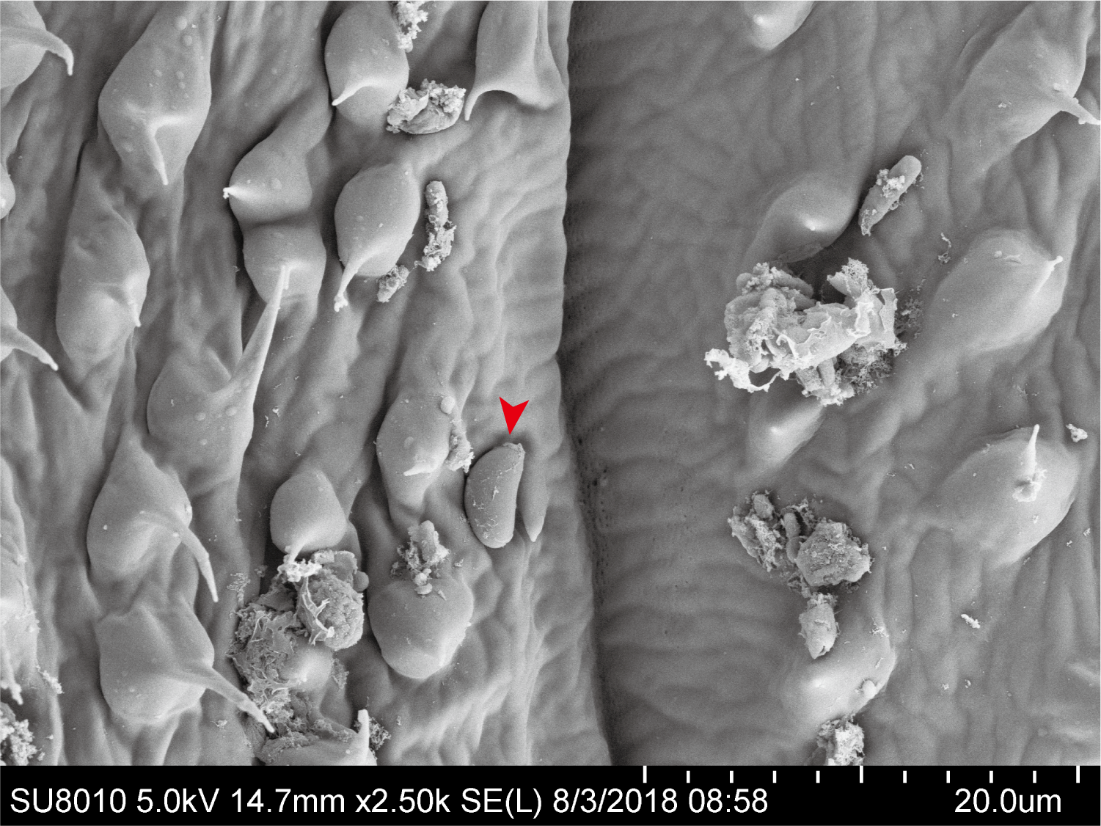
**

**Supplementary Figure 1.** Scanning electron micrographs of *Thitarodes xiaojinensis* larval epicuticle after inoculation with *Ophiocordyceps sinensis* conidia. *O. sinensis* conidium (red arrowhead) was found on the cuticle surface, but differentiated appressoria or hyphae were not found.

**
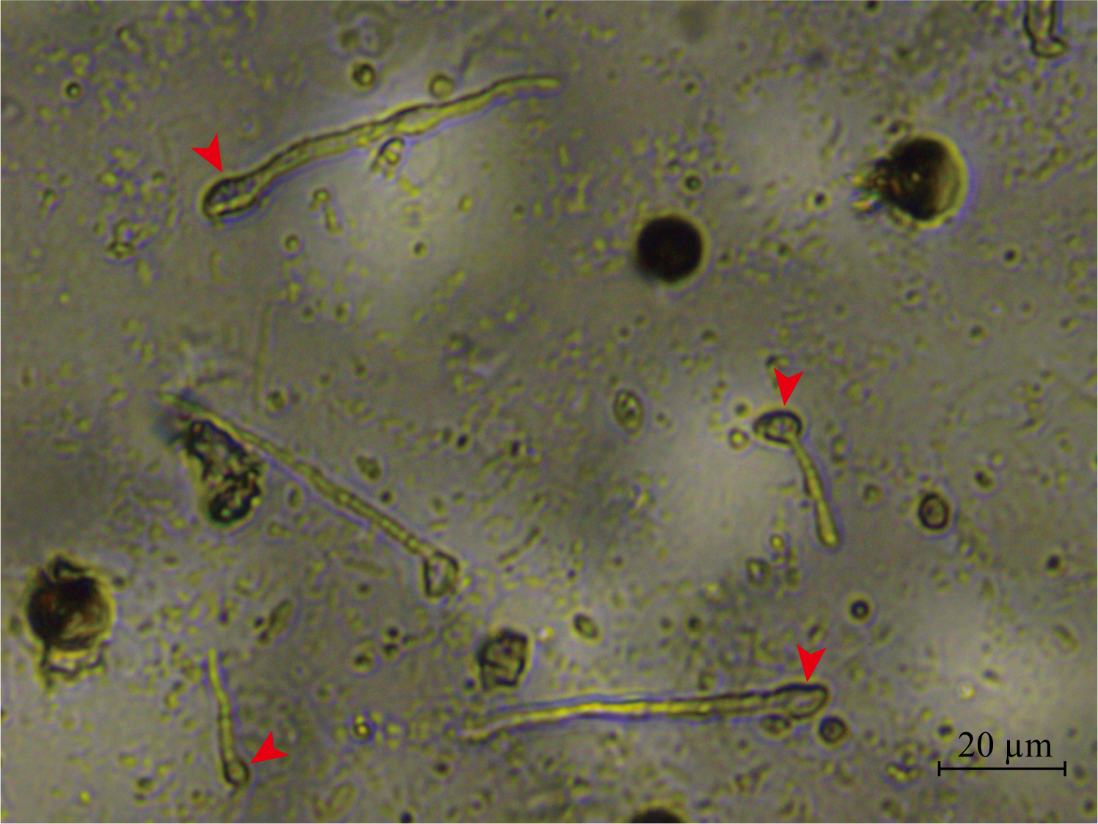
**

**Supplementary Figure 2.** *Ophiocordyceps sinensis* conidia germinated on locust wings, and appressoria were not formed. The red arrowhead indicates germinated conidia.

# Supplementary Tables

**Supplementary Table 1.** Primers used in plasmid construction.

| **Experiment** | **Fragment** | **Primer (5'–3')** | |
| --- | --- | --- | --- |
| Construction of Blunt-GFPT plasmid by blunt-ended cloning method | EGFP-NOS | Forward | ATGGTGAGCAAGGGCGAGG |
|  |  | Reverse | GATCTAGTAACATAGATGACACCGC |
| Constructing of Blunt-PHPHT plasmid by blunt-ended cloning method | EcoRI-PtrpC-HPH-CaMV | Forward | GAATTCGTAATCATGGTCATAGCTG |
|  |  | Reverse | GACAACTTAATAACACATTGCGGAC |
| Construction Blunt-OsPEF1GFPT plasmid by FastCloning method | EcoRI-OsPEF1α | Forward | accatgattacgaattcGCTCAAGTTGGGCATGACGGGACCC |
|  |  | Reverse | tcgcccttgctcaccatCTTGGCGAGGTTTGTGGAGGTGAAG |
|  | Linear Blunt-EGFP-NOS | Forward | ATGGTGAGCAAGGGCGAGGAG |
|  |  | Reverse | GAATTCGTAATCATGGTCATAGCTG |
| Construction pBHt2-OsPEF1α-GFP plasmid by FastCloning method | HPH-EGFP-BOX | Forward | aaacaaattgacgcttaGACAACTTAATAACACATTGCGGACG |
|  |  | Reverse | gccagtgccaagcttCCTCTAGATGCATGCTCGAGC |
|  | Linear pBHt | Forward | AAGCTTGGCACTGGCCGTCGTTTTACAAC |
|  |  | Reverse | TAAGCGTCAATTTGTTTACACCACAATATATCCTGCC |

The underline indicates the restriction enzyme site. Lowercase letters indicate the additional overlap of insert fragment with plasmid.

**Supplementary Table 2.** The numbers of key genes involved in melanin synthesis in different entomopathogenic fungi.

| **Gene** | ***Ophiocordyceps sinensis*** | | ***Cordyceps militaris*** | ***Beauveria bassiana*** | ***Metarhizium robertsii*** |
| --- | --- | --- | --- | --- | --- |
| Tyrosinase | | 9 | 6 | 6 | 7 |
| Protein tyrosine phosphatase | | 3 | 2 | 2 | 1 |
| Chorismate mutase | | 2 | 2 | 1 | 4 |
| Protein tyrosine kinase | | 2 | 0 | 1 | 0 |
| Polyketide synthases | | 31 | 10 | 14 | 21 |
| Peroxidase | | 19 | 5 | 5 | 11 |
| Phenylalanyl-tRNA synthetase | | 3 | 3 | 2 | 3 |
| Phenol hydroxylase | | 3 | 0 | 1 | 0 |
| Aromatic-L-amino-acid decarboxylase | | 1 | 1 | 0 | 0 |
| Glyceraldehyde-3-phosphate dehydrogenase | | 1 | 0 | 1 | 0 |

The data were derived from published genomics studies.
